# The Multiple Correspondence Analysis Method and Brain Functional Connectivity: Its Application to the Study of the Non-linear Relationships of Motor Cortex and Basal Ganglia

**DOI:** 10.3389/fnins.2017.00345

**Published:** 2017-06-20

**Authors:** Clara Rodriguez-Sabate, Ingrid Morales, Alberto Sanchez, Manuel Rodriguez

**Affiliations:** ^1^Laboratory of Neurobiology and Experimental Neurology, Department of Physiology, Faculty of Medicine, University of La LagunaTenerife, Spain; ^2^Centro de Investigación Biomédica en Red sobre Enfermedades NeurodegenerativasTenerife, Spain; ^3^Department of Pharmacology and Physical Medicine, Faculty of Medicine, University of La LagunaTenerife, Spain

**Keywords:** multiple correspondence analysis, functional connectivity, basal ganglia, cerebral cortex, resting state

## Abstract

The complexity of basal ganglia (BG) interactions is often condensed into simple models mainly based on animal data and that present BG in closed-loop cortico-subcortical circuits of excitatory/inhibitory pathways which analyze the incoming cortical data and return the processed information to the cortex. This study was aimed at identifying functional relationships in the BG motor-loop of 24 healthy-subjects who provided written, informed consent and whose BOLD-activity was recorded by MRI methods. The analysis of the functional interaction between these centers by correlation techniques and multiple linear regression showed non-linear relationships which cannot be suitably addressed with these methods. The multiple correspondence analysis (MCA), an unsupervised multivariable procedure which can identify non-linear interactions, was used to study the functional connectivity of BG when subjects were at rest. Linear methods showed different functional interactions expected according to current BG models. MCA showed additional functional interactions which were not evident when using lineal methods. Seven functional configurations of BG were identified with MCA, two involving the primary motor and somatosensory cortex, one involving the deepest BG (external-internal globus pallidum, subthalamic nucleus and substantia nigral), one with the input-output BG centers (putamen and motor thalamus), two linking the input-output centers with other BG (external pallidum and subthalamic nucleus), and one linking the external pallidum and the substantia nigral. The results provide evidence that the non-linear MCA and linear methods are complementary and should be best used in conjunction to more fully understand the nature of functional connectivity of brain centers.

## Introduction

**Figure 1:**
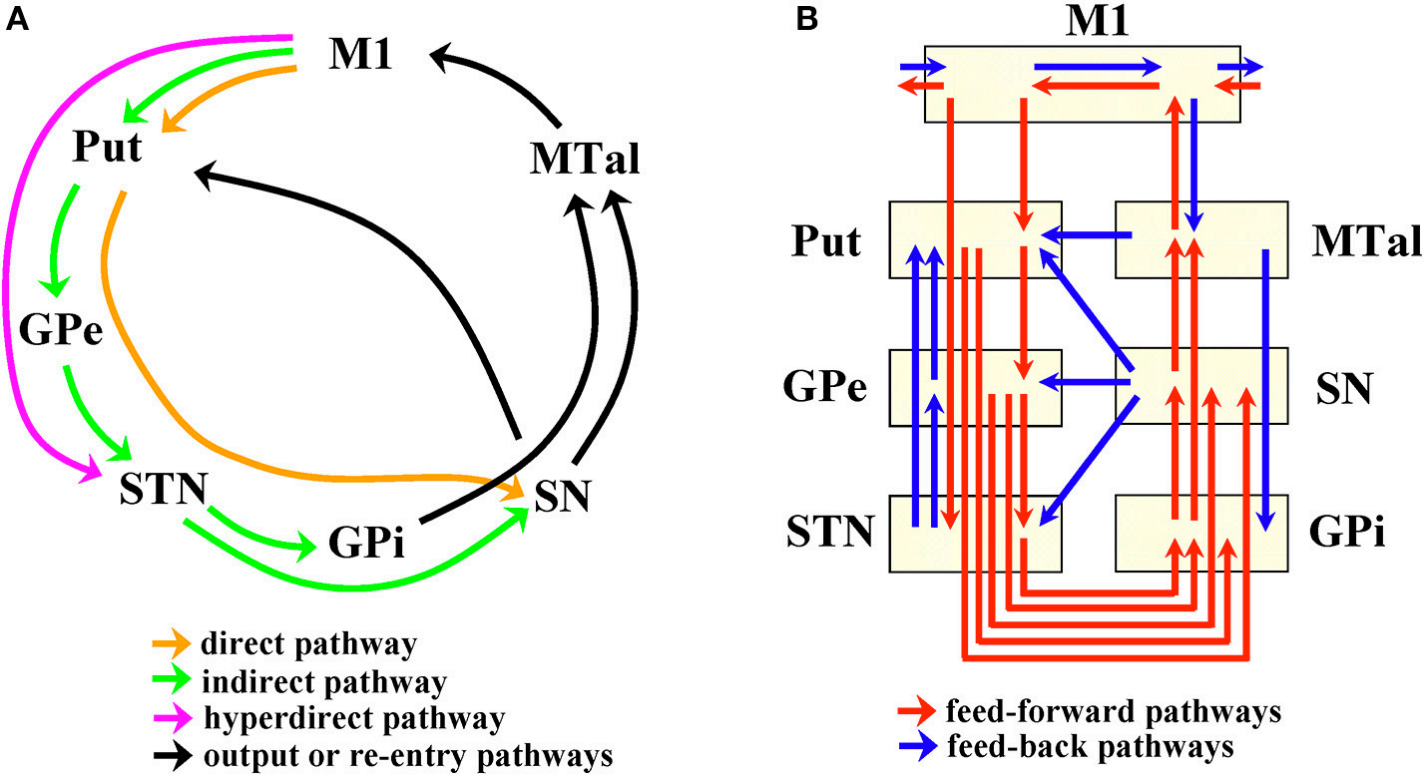
Connectivity of basal ganglia according to the cortico-subcortical loop **(A)** and to feedforward-feedback pathways **(B)**. M1, primary motor cortex; S1 somatosensory cortex; Put, putamen; GPe, external globus pallidumN; STN, subthalamic nucleus; GPi, internal globus pallidum; SN, substantia nigral; MTal, motor thalamus; MTal.

Basal ganglia (BG) are composed of a number of interconnected subcortical centers which receive projections from all cortical areas and return the processed information to its cortical origin. The global dynamic of BG is currently estimated based on the local excitatory/inhibitory interactions of their main centers, which is a useful strategy to explain some of the functional disturbances of Parkinson's disease (PD) and other frequent neurological disorders. However, closed-loop circuits may have complex dynamics whose emergence cannot be extrapolated from the local interactions of their components. Current models include five parallel cortico-subcortical closed-loops linking the activity of BG to those of the brain cortex. One of these cortico-subcortical circuits is the “motor loop,” which is a circuit composed of neurons projecting from the primary motor (M1) and somatosensory (S1) cortex to the caudate and putamen (Put), and from these centers to the external globus pallidum (GPe), subthalamic nucleus (STN), internal globus pallidum (GPi), and substantia nigral (SN). Motor information processed by these centers goes to the anterior thalamus (motor thalamus; MTal) and then returns to M1/S1. The “motor loop” is composed of three main components: the direct pathway (M1-Put-SN/GPi-MTal-M1), the indirect pathway (M1-Put-GPe-STN-GPi/SN-MTal-M1), and the hyperdirect pathway (M1-STN-SN/GPi-MTal-M1) (Figure 1A). These feed-forward circuits have been widely used over the last 20 years to explain different movement disorders and to justify the beneficial effects of drugs and surgical therapies in PD (Alexander et al., 1986; Penney and Young, 1986; Albin et al., 1989; Delong, 1990; Obeso et al., 2008a,b). However, the finding of a number of subcortical loops and feed-back circuits (Redgrave et al., 1992; Mchaffie et al., 2005) suggests that the dynamic for BG could be more complex than that expected for these feed-forward circuits (Figure 1B). In addition, BG interactions may be mediated by “crossing centers” which transfer but do not process information, thus facilitating the functional interactions of some centers of the closed-loop circuit which do not have direct structural interconnections (Rodriguez-Sabate et al., 2015).

Methodological restrictions currently limit the study of the BG dynamic in humans, with human models of BG being mainly based on animal data. The most commonly methods used to study human BG are based on recording the blood-oxygen-level-dependent (BOLD) signal by magnetic resonance. Functional magnetic resonance imaging (fMRI) methods use the increase of the BOLD-signal level to distinguish the BG involved in particular tasks (centers showing a BOLD-level increase during the execution of a task are generally considered as being involved in the task execution). However, fMRI does not provide information about the functional interaction between centers. This interaction is better approached by studying the temporal relationship of the spontaneous fluctuation of their BOLD-signals (functional connectivity MRI; **fcMRI**) (Fox and Raichle, 1986; Fox et al., 1988; Kim and Ugurbil, 1997; Raichle, 1998; Van Dijk et al., 2010). fcMRI has some methodological restrictions which can be particularly challenging for BG studies. The limited spatial-resolution of fcMRI hampers the finding of BOLD-signals representing the smallest BG. This weakness can be counteracted by: (1) associating the BOLD-images to high spatial-resolution anatomical images (recorded in the same session and with the head fixed to the coil), (2) working with BOLD-signals computed from regions of interest (ROIs) located in the central portion of each nucleus of each individual (instead of working with data obtained after averaging functional images of different subjects), and (3) manually identifying each BG on a subject-by-subject basis (3D-anatomical images) and using both internal (e.g., the shape of the nucleus in the high-resolution MRI) and external (e.g., the anatomical relationship of the nucleus with neighboring structures) cues. Under these conditions, the contamination of the BOLD-signal of a center by those from the neighboring BG may be controlled (Rodriguez-Sabate et al., 2015). The relatively low time-resolution is another limitation of fcMRI. The excitatory/inhibitory interactions of BG (which generally occur with latencies lower than 0.1 s) cannot be properly identified by fcMRI (whose time-resolution is over 1 s). However, the time-resolution of fcMRI is enough to study multisecond BOLD-oscillations which also provide information about the dynamic of large-scale functional networks (Filippov, 2005; Fox and Raichle, 2007; Fox et al., 2009; Rayshubskiy et al., 2014). The analysis of these very-slow fluctuations has been mainly performed by correlating BOLD-signals recorded from different centers, with the Pearson correlation being the most widely used procedure (Biswal et al., 1995; Fox and Raichle, 2007; Fox et al., 2007; Di Martino et al., 2008; Treserras et al., 2009; Cole et al., 2010). The efficacy of the Pearson correlation decreases in centers with non-linear relationships, a problem which is less relevant for other methods such as the non-parametric Spearman correlation, partial correlation coefficient, mutual information, wavelet correlation coefficient, extended maximal information coefficient, and coherence methods (Reshef et al., 2011; Su et al., 2013; Kinney and Atwal, 2014). However, these methods include centers in a network when they show any relationship with a particular nucleus selected as a “seed” region. Thus, they perform pair-wise associations (between the “seed” center and potential candidates to be included in the network) but not multi-link associations (between three or more centers). This fact decreases the sensitivity of these methods to identify networks, particularly when they are composed of centers which are massively interconnected by non-linear feed-back interactions. In addition, they are hypothesis-driven methods which will not identify networks not predicted by the examiner. The multiple linear regression may be used to predict the BOLD-activity of a center (dependent variable) as a result of the BOLD-activity of other centers selected as predictors (independent variables). However, this is also a hypothesis-driven method which is less sensitive with non-linear relationships (non-linear regression methods such as the generalized linear/non-linear models, multivariate adaptive regression splines, or the structural equation modeling present complex interpretations and need of hypothesis-driven procedures). On the other hand, there are multivariate methods which can be used to identify massive interacting networks with “unsupervised” data-driven procedures. This is the case of the independent component analysis (ICA), a method that induces a self-organizing clustering of centers according to their interactions, and which has proved useful to identify cortical networks (Mckeown and Sejnowski, 1998; Damoiseaux et al., 2006; Goebel et al., 2006; Fox and Raichle, 2007; Li et al., 2009; Smith et al., 2009). However, this procedure assumes there are linear interactions between the centers of the network, a fact that, as will be shown below, is not very common in human BG. An alternative to ICA is the data-driven sparse GLM, which extracts individually adaptive activation patterns more accurately than spatial and temporal ICA (Lee et al., 2011; Su et al., 2016). The multiple correspondence analysis (MCA) is introduced here, which is a data-driven method which can be used to identify non-linear interactions in categorical data. Although MCA has been successfully implemented in different disciplines including psychology and health sciences (Bouilland and Loslever, 1998; Guinot et al., 2002; Meyer et al., 2004; Ambrogi et al., 2005; Almeida et al., 2009; Rennie and Roberts, 2009; Pinti et al., 2010; Avolio et al., 2013; Costa et al., 2013; Sagawa et al., 2013; Ayele et al., 2014; Touso et al., 2014), it is seldom encountered in neuroimaging literature. This method was applied here to study the functional connectivity of BG, centers which have shown multiple non-linear interactions in both animal and human studies (Rodriguez et al., 2003a,b; Marceglia et al., 2006; Schroll and Hamker, 2013).

The initial hypothesis of this work was that the motor-loop of BG contains several functional networks which may be identified by studying the time relationship of the slow BOLD-fluctuations of their main centers with MCA. The first step was to categorize each BOLD-data as high or low level (according to its mean value), thus replacing the analogical BOLD-data by discrete data representing the status of each center (high activity vs. low activity). MCA showed BG co-activations of the different BG, revealing functional configurations which were not detected by pair-wise comparisons (Pearson and Spearman correlations) or multiple regression analysis. Finally, the relationship between centers grouped by the MCA was studied with the correspondence coefficient (CC), an index of co-activation degree between individual centers of a network, and which was computed from the Burt table of relative frequencies.

## Methods

### Participants

Twenty four right-handed volunteers (12 males and 12 females 24–64 years of age) with no history of neurological or mental diseases were included in the study (Oldfield, 1971). MRI studies were performed following recently reported methods (Rodriguez-Sabate et al., 2015). All procedures were in accordance with the ethical standards of the 1964 Helsinki declaration, and performed with the approval of the local Institutional Human Studies Committee of La Laguna University. All individual participants provided written, informed consent.

### MRI recording

The involuntary movement of the head during the MRI studies was prevented by attaching the head to the fixed head-coil of the MRI equipment. BOLD contrast images (64 × 64 sampling matrix with brain slices 4-mm thick and 4 × 4 mm voxels in-plane resolution) were acquired (General Electric Medical System 3.0 T) in a coronal plane (250 × 250 mm field of view) with gradient-echo (echo-planar imaging with a repetition time of 1,600 ms; and echo time of 21.6 ms; a flip angle of 90°). Data were recorded with subjects at rest (the instruction was “Stop moving and do not make any particular action”). 180 volumes were recorded in each subject under these conditions. fMRI data were co-registered with 3D anatomical images (repetition time 7.6 ms; echo time 1.6 ms; flip angle 12°; 250 × 250 mm field of view; 256 × 256 sampling matrix; 1 mm slice thickness and 1 x 1 mm voxel resolution). A balanced position between the space vs. time resolution was adapted here for fcMRI studies to prevent a high spatial resolution from possibly decreasing the time resolution (1.6 s in this study) and hampering the identification of functional interactions between BG. As space resolution affects the construction of ROIs representing the activity of the smallest BG (e.g., STN), special care was taken to place each ROI in each subject. Based on a previous work (Rodriguez-Sabate et al., 2015), ROIs (4 mm isotropic) were located on anatomical studies (1 mm isotropic voxels) performed in the same session, with a stable head position so that the correspondence between the structural and functional images was not affected. Different structural markers were jointly used to place each ROI, particularly in the case of the smallest BG. The volume of the smallest BG is low, particularly in the case of the subthalamic nucleus. The spatial resolution of the BOLD signals can be increased by decreasing their time-resolution, a fact which produces problems in functional connectivity studies. Thus, we opted for a workable compromise which includes a spatial resolution of 4 mm and a time resolution of 1.6 s. Of course, it is not possible to rule out the possible influence of a partial volume effect which could underestimate the activity of the smallest centers (such as the subthalamic nucleus) on the BG dynamic. The functional and anatomical studies were always obtained in a single session and with the head fixed in the same position of the field-of-view to facilitate a stable relationship between each fcMRI voxel (4 × 4 × 4 mm) with the corresponding 64 structural voxels (1 × 1 × 1 mm) during the whole study. All data sets were normalized to the Talairach space.

### Data preprocessing

The preprocessing of data (performed with the BrainVoyager software; version 2.1.2 of BVQX) included a slice scan time correction (cubic spline), a 3D motion correction (trilinear interpolation), and a temporal filtering (high-pass GLM-Fourier filter which removed frequencies below 0.009 Hz). Unwanted BOLD-correlations produced by coherent fluctuations originated from residual motion artifacts and physiological signals were prevented both by restraining the head movements during the BOLD-signal recording and by “regressing” the recorded BOLD time-series with “regressors” computed from BOLD signals simultaneously recorded in the white matter and brain ventricles (which are largely independent of the neural activity but which are sensitive to confounding variables including scanner instabilities, subject motion, respiration, cardiac effects) (Murphy et al., 2009; Anderson et al., 2011; Saad et al., 2012; Jo et al., 2013; Power et al., 2014).

### Identification of representative ROIs of basal ganglia

The spatial resolution was established at 4 mm (a higher spatial resolution induced an undesirable increase of the repetition time over 1.6 s), in order to reach the time resolution needed to study the functional connectivity between BG. Although this is not a high spatial resolution, it proved to be high enough to study the smallest BG. According to previously reported methods (Rodriguez-Sabate et al., 2015), particular attention was paid to identifying representative ROIs of the smallest BG, which were located on a subject-by-subject basis by considering (1) the Talairach coordinates, (2) the shape of the nucleus, and (3) the anatomical relationship of the nucleus with neighboring structures. All centers were identified in coronal slices located 4–27 mm posterior to the anterior commissure (Table 1). **GPi** was identified ≈6 mm posterior to AC and just above the optic tract. The **putamen** zone which receives projections from the somato-sensorimotor cortex was identified in the postcomisural region (≈5 mm posterior to AC) (Selemon and Goldman-Rakic, 1985; Parent, 1990; Haber, 2003; Nambu, 2011). **GPe** was located ≈3 mm posterior to AC and **MTal** was located ≈11 mm posterior to AC (5 mm posterior to the GPi). The **STN** was identified in the slice where the oculomotor nerve was trapped in the most medial region of the contact between the pons and cerebral peduncle, just above a horizontal line crossing the optic tract (10 mm medial to this tract), and near the medial boundary of the cerebral peduncle (the STN ROI was small and clearly located within the nucleus). **SN** pars compacta is intermixed with SN pars reticulata in humans and both portions of the SN cannot be clearly segregated in MRI images. Thus, the ROI of this center included the whole SN, which was located between the red nucleus and the cerebral peduncle (22–26 mm posterior to AC). The **M1** was located in the precentral gyrus posterior to the junction of the superior frontal sulcus with the precentral sulcus, and according to a previously reported procedure (Rodriguez et al., 2004). Additional comments about the ROI identification can be found in previous studies (Rodriguez et al., 2004; Rodriguez-Sabate et al., 2015, 2016).

**Table 1:** Coordinates (normalized to Talairach) and size (number of voxels) of ROIs of the centers included in the study.

	***X***	***Y***	***Z***	**Size**
Primary somatosensory cortex	36.0 ± 8.9	−26.7 ± 3.7	51.3 ± 8.0	41.5 ± 12.1
Primary motor cortex	38.4 ± 6.3	−20.3 ± 5.3	49.2 ± 7.1	36.8 ± 11.1
Putamen	27.5 ± 1.5	−5.4 ± 1.5	0.1 ± 0.3	22.8 ± 3.9
External pallidum	15.8 ± 4.8	−2.4 ± 1.1	2.7 ± 2.2	11.2 ± 2.8
Internal pallidum	14.4 ± 2.1	−6.4 ± 1.2	−1.7 ± 1.8	12.2 ± 1.9
Subthalamic nucleus	10.8 ± 1.7	−13.7 ± 2.2	−4.4 ± 2.8	2.3 ± 0.4
Substantia nigra	7.3 ± 1.2	−18.9 ± 1.3	−8.4 ± 2.6	256.2 ± 31.1
Ventral-anterior thalamus	9.5 ± 1.1	−11.2 ± 1.2	7.2 ± 2.4	29.6 ± 8.8

*Values are mean ± standard error*.

### Correlation and multiple linear regression methods

All the voxels of ROIs of the same center in the right and left brain sides were grouped together (Gopinath et al., 2011), and BOLD-values were then normalized according to their mean value (BOLD-value ^*^ 100/mean BOLD-value computed for the whole recording). Thus, the normalized BOLD-signals fluctuated around the value 100 in all centers (which facilitates the data analysis with MCA and the other methods). These normalized values were used to compute the Pearson (**r**) and Spearman (**S**) correlations and the multiple linear regression. The functional relationship between each center and all the other BG was initially estimated with multiple linear regression, including the computation of the **regression coefficient (B**; partial correlation of each independent variable with the dependent variable), the **correlation coefficient (R**; the degree to which the independent variables predict the dependent variable), and the **coefficient of determination (R**^2^; an indicator of how well the model fits the data). The statistical significance for these statistics was computed with a two-tailed *t*-test (*p* < 0.001).

### Multiple correspondence analysis (MCA) and correspondence coefficient (CC)

The MCA is a multivariate method (Caceres et al., 2010; Pinti et al., 2010) (Greenacre, 1992, 2010; Grassi and Visentin, 1994) which distributes values of a table of relative frequency (Burt table) in an n-dimensional space, and then uses the distance between the variables in each dimension to establish the similarity degree of variables. Applied to BOLD-signals representing the activity of the main BG, MCA was used here to perform an unsupervised (driven by data) identification of centers with a similar functional connectivity (those showing co-activation). Thus, MCA is free from assumptions and, working with categorical data, it may represent linear and non-linear relationships equally well. These are key advantages over more traditional techniques which may need linear relationships between variables and a previous hypothesis about the possible variable relationships. MCA is not very familiar in the neuroimaging world and, as far as we know, it has never been used to analyze fMRI data. Therefore, the MCA terminology used in this study is defined in the following paragraph, and it can also be consulted in different reports (Greenacre, 1992; Grassi and Visentin, 1994; Greenacre, 2010).

**Figure 2:**
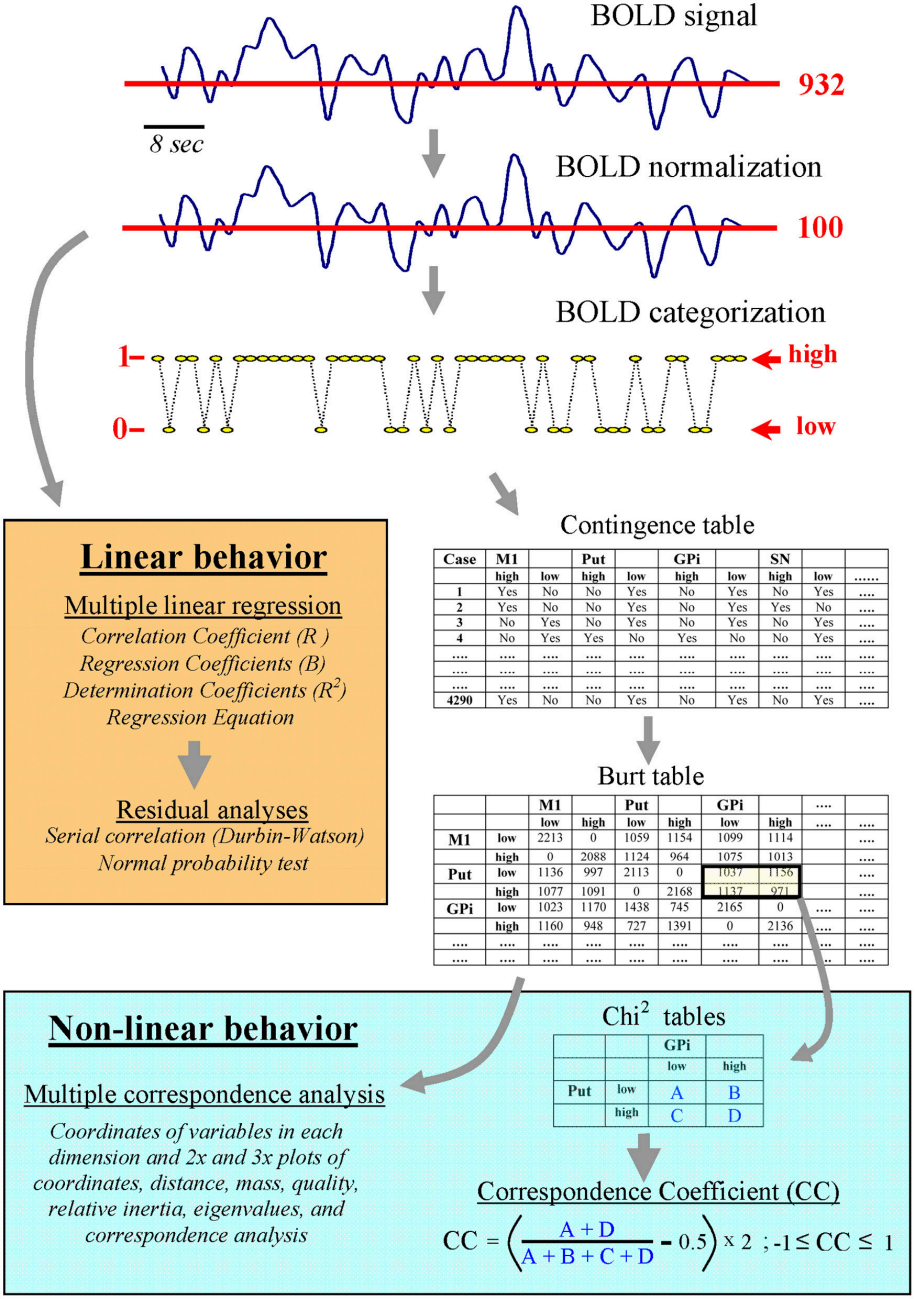
Linear and non-linear computing methods for analyzing the normalized and the categorized BOLD-signal.

Because MCA works with categorical data, the first step was to categorize the previously normalized BOLD-data (see above and Figure 2). Bearing in mind that the normalized BOLD-signals fluctuate around the value 100 (which is the mean value of the normalized BOLD-signals), the categorization of signals was performed by replacing the normalized BOLD-data with the number 1 (high status) if they were higher than 100, and with the number 0 (low status) if they were lower or equal to 100 (categorized BOLD-signal). The second step was to compute a contingency table using categorized data. This table had two columns per BG, one for the low status and the other for the high status. There was a row for each of the BOLD-points recorded in the BG of each subject (180 BOLD-points × 8 centers × 24 subjects). Thus, the rows represented the relative status (high-low) of each center in a particular moment of the MRI recording. Finally, the frequency of inter-center co-activations was grouped in the Burt table, which displayed the number of low-low, high-high, high-low and low-high coincidences between the different BG. This table was used to compute MCA.

MCA used the frequency distribution provided by the Burt table to distribute all variables across each of the computed dimensions (7 dimensions were chosen in the present study), with variables with the lowest distance being considered as those with the highest degree of similarity in the corresponding dimension. The interpretation of the possible meaning of each dimension was performed using the outliers (variables that contribute most to the information of a dimension), a possibility that will be commented on below. The distance between coordinates in the MCA space was initially computed in a Euclidean metric and then normalized to a Chi^2^ metric whose values ranged between −1 to +1. Two centers showing similar Chi^2^ coordinates indicate that they have analogous interactions with the other BG (a similar probability of high/low status with the other BG centers). In this way, the MCA grouped centers according to their functional connectivity with the other BG (global similarities), a possibility which cannot be performed by analyzing series of pair-wise correlations or by using the multiple regression analysis. The MCA is similar to the principal component analysis but with the advantage that it can be suitably applied to categorized data obtained from brain centers with non-linear relationships.

The validity of the MCA was evaluated by different complementary approaches. The different MCA metric considered were:

- **Inertia** which shows the dispersion of data around their center of gravity G (or centroid) and which is considered as a measure of information. The term inertia is used by analogy with the definition in applied mathematics of “moment of inertia,” which stands for the integral of mass times the squared distance of the centroids.
- **Total inertia** which represents the inertia of all BG in all the dimensions analyzed (it was normalized between 0 -no information- and 1 -all available information-).
- **Accumulated inertia** which shows the inertia of each dimension (all the centers grouped together) accumulated with the inertia of the lower dimensions. The accumulated inertia of the highest dimension is the same as the total inertia. Starting from a 16 × 16 Burt table (8 centers -BG- × 2 states -high and low-), 16 dimensions could be computed (no. columns -1 + no. of rows- 1). The aim of MCA is to decrease the number of dimensions in a manner that retains almost all information (in other words to reproduce the distances between the row and/or column points of the Burt table in a lower-dimensional space). The accumulated inertia was used to select the minimum number of dimensions necessary to include almost all the information of the time-series (7 dimensions covered ≈95% of information in this study).
- **Relative inertia** represents the inertia of each variable in each dimension (normalized between 0 and 1 which represents all the information of a variable in all the dimensions). The relative inertia has also been referred to as “contribution” in MCA literature.
- The **quality of a variable** (**cosine**^2^) represents the distribution of the inertia of this variable across dimensions (normalized between 0 and 1 which represents whole inertia of the variable; a value lower than 0.1 indicates a poor representation of the variable in the computed dimensions). The term cosine^2^ refers to the fact that this value is also the squared cosine value of the angle the point makes with the specific dimension (it may also be interpreted as the correlation of the respective point with the respective dimension).
- **Eigenvalues** represent the relative relevance of each dimension to the total inertia (it is normalized to 1 which represents all the information of all the variables in all the dimensions). The highest eigenvalue was always in the first dimension, progressively decreasing across the following dimensions. This variable (together with the accumulated inertia) is commonly used to select the maximum number of dimensions to be included in the MCA. Thus, dimensions with an eigenvalue lower than 0.05 are frequently not considered.
- **Outliers** are the variables (BG) that contribute most to the information of each dimension, Outliers were detected by identifying BG with the highest coordinate values and a high contribution to the dimension (a high variable quality and relative inertia). Outliers were used to facilitate the interpretation of the possible meaning of the dimension.

The MCA computation was performed by the Statistics program (StatSoft, Tulsa). MCA can identify the centers of a network (those with a similar functional connectivity), but it does not provide information about the possible interaction between these centers. The introduction of individual BG as supplementary points of the MCA could be used to estimate intra-network relationships but, after testing this procedure, it did not show enough clear data to be included in this study. Thus, a procedure which used the Burt table (bottom-right Figure 2) to estimate the co-activation probability of particular BG was introduced (**correspondence coefficient**; CC). CC represents the coincidence degree (high-high plus low-low states) vs. anti-coincidence degree (high-low plus low-high states) of the activity of two centers. Data were normalized in such a way that the CC was always between +1 and −1. CC values near +1 indicate a marked co-activation (coincidence of their low-low and high-high status), and values near −1 indicate a frequent anti-coincidence (when one center was in a high status the other was in a low status and vice versa). CC values near 0 indicate that the status of two BG centers have a random relationship (points are randomly distributed in the four squares). CC is similar to the Phi coefficient (also known as the correlation coefficient of Mathews) but applied to data of the Burt table. The statistical significance of the CC was estimated by the Chi^2^ test of independence.

## Results

**Figure 3:**
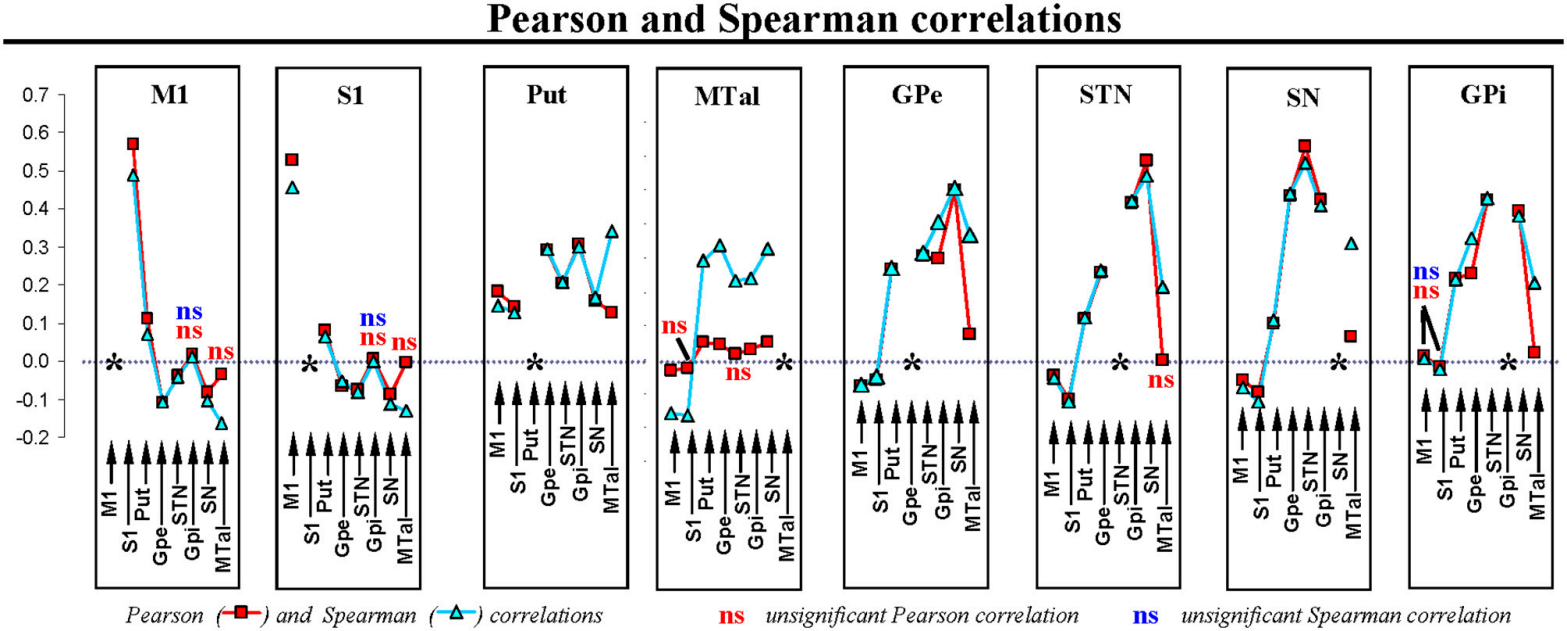
Pearson and Spearman correlation coefficients for the individual interactions of BG. M1, primary motor cortex; S1, somatosensory cortex; Put, putamen; GPe, external globus pallidumN; STN, subthalamic nucleus; GPi, internal globus pallidum; SN, substantia nigral; MTal, motor thalamus; MTal.

Table 1 shows the position and size (no. voxels) of ROIs used to characterize the BOLD activity. Figure 3 shows the Pearson and Spearman correlations between BG. In this figure, all correlations showed statistical significance except for those indicated by the letters “ns.” Thus, M1 and S1 showed a marked positive correlation between each other and a more moderate positive correlation with the putamen. M1 and S1 showed a negative correlation with GPe, STN, and SN. Put showed a positive correlation with all BG, and with M1 and S1. MTal showed a positive correlation with BG and a negative correlation with M1 and S1 (the Pearson correlation with S1 and STN did not show statistical value). GPe, STN, and SN showed a positive correlation between one another and a negative correlation with M1 and S1. GPi showed a positive correlation with the other BG but not with M1 and S1.

**Figure 4:**
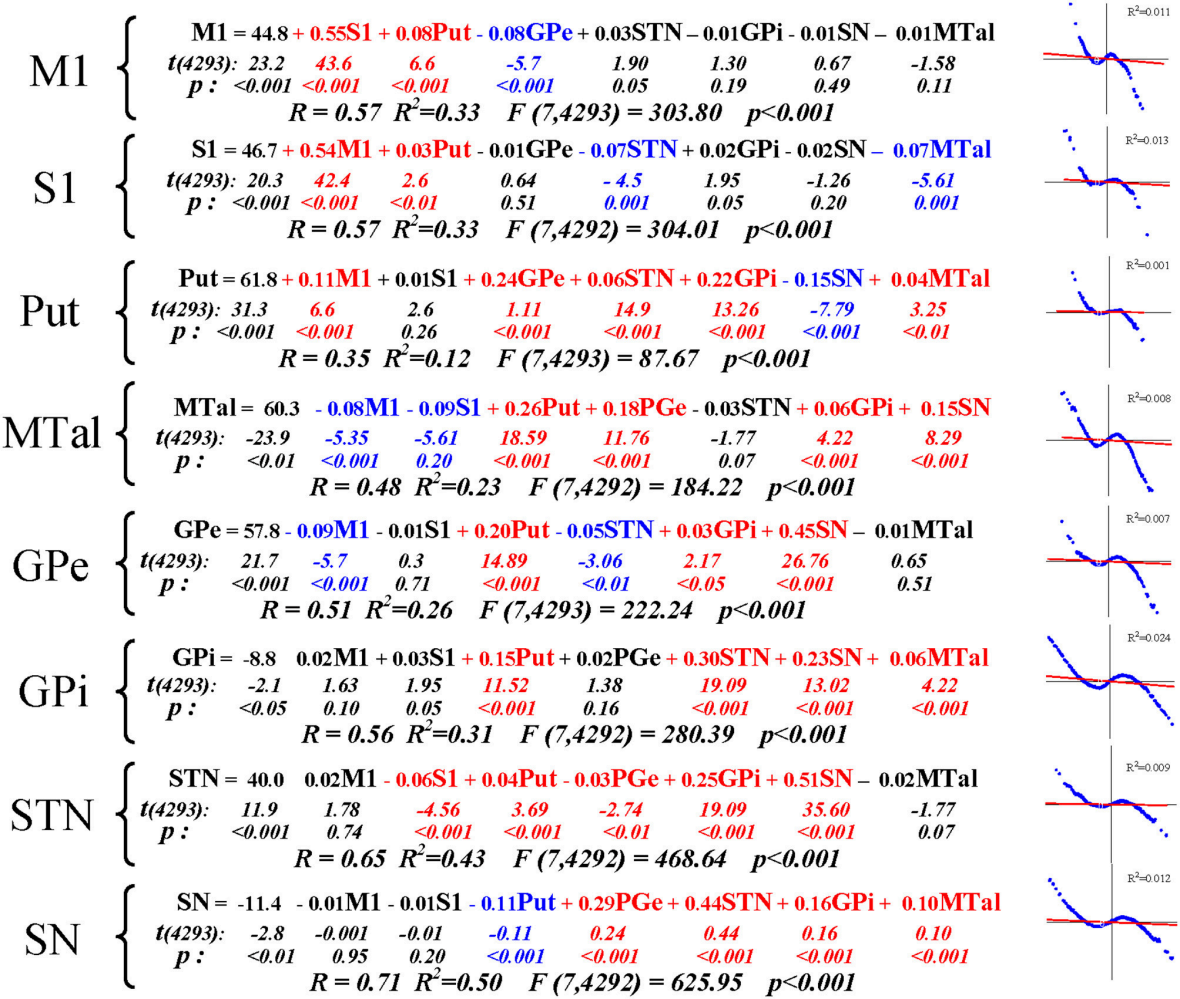
Multiple regression analysis of BG interactions. The distribution of residuals (values predicted by the regression equation minus observed values) regarding values expected according to a normal distribution of residuals (red line) are shown on the right of each regression equation. M1, primary motor cortex; S1, somatosensory cortex; Put, putamen; GPe, external globus pallidumN; STN, subthalamic nucleus; GPi, internal globus pallidum; SN, substantia nigral; MTal, motor thalamus; MTal; R, correlation coefficient; R^2^, coefficient of determination.

Figure 4 shows the multiple linear regressions of BG, using one center as dependent variable and the other centers as independent variables. All the regression equations showed a significant statistical value, with a correlation coefficient R higher than 0.35 and a coefficient of determination R^2^ higher than 0.12. The regression coefficient B showed a significant positive partial correlation between M1, S1 and Put (see the first three equations at the top of Figure 4). Negative partial correlations were observed between M1-GPe and between S1-STN and S1-MTal. Put showed a positive relationship with M1, GPe, STN, GPi, and MTal, and a negative relationship with SN. MTal showed a positive relationship with Put, GPe, GPi, and SN, and a negative relationship with M1 and S1. GPe showed a positive relationship with Put, GPi, and STN, and a negative relationship with M1 and STN. GPi showed a positive relationship with Put, STN, SN, and MTal. STN showed a positive relationship with S1, Put, GPe, GPi, and SN. SN showed a positive relationship with GPe, STN, GPi, and MTal, and a negative relationship with Put. The analysis of the residuals of the regression equations (predicted minus observed values) did not show the expected normal distribution. This fact can be observed in images included on the right of the equations in Figure 4, Q-Q plots where the expected normal distribution (plotted as a red line; Y = X line) and the distribution of residuals (plotted as blue points) show clear disagreements. This fact, and the curvature of the distribution of residuals, show non-linear components in the relationship between most BG. Even under these circumstances, equations of the multiple linear regression proved to be suitable for predicting the BOLD-behavior of centers (used as dependent variables) with the BOLD-behavior of the other centers (used as independent variables). However, multiple linear regression does not provide information about the underlying causal mechanisms (e.g., excitatory/inhibitory interactions between centers), or about multiple interactions between several centers (coefficient B of the regression equation represents the partial correlation of the corresponding independent variable with the dependent variable). MCA, a data-driven method which groups BG according to their co-activations, identified multiple interactions better.

The MCA identified seven subgroups of BG with similar functional interactions which could be referred to by different generic names, including “functional network” and “functional configuration.” Although the term “functional network” has been commonly used for centers grouped by other fcMRI methods in the brain cortex, it may not be the most appropriate term in the present context. “Functional network” could suggest that centers are wired by selective pathways, which is not necessarily true because they can be “indirectly” linked by other centers, which is more probable in centers which, as occurs in BG, are included in closed-loop circuits with multiple feed-back interactions (Figure 1B). In addition, the name “functional network” may suggest that all centers are normally recruited at the same time by in-phase fluctuation, which was not always the case here. The term “functional configuration” (fc) which does not suggest either direct wiring or simultaneous in-phase recruitment of centers (a configuration may present both in-phase and anti-phase association of their high vs. low status) will be used here to refer to the BG subgroups identified by the MCA.

**Figure 5:**
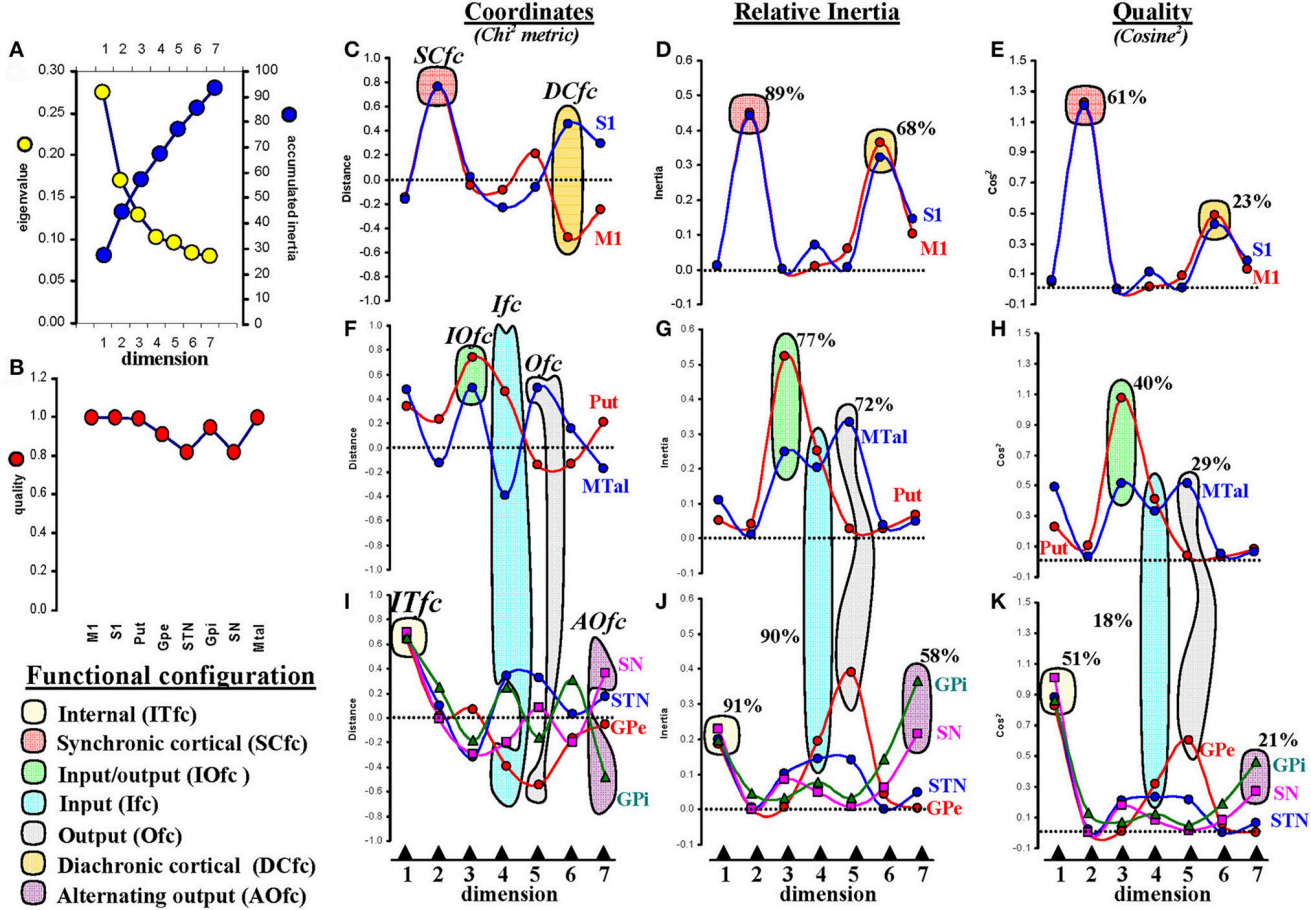
Multiple correspondence analysis (MCA). **(A)** the eigenvalue (contribution of each dimension to the total inertia; it is normalized to 1 which represents all the information of all the variables in all the dimensions) and accumulated inertia (inertia of each dimension added to those of lower dimensions; it is normalized to 100 which represents the total inertia of all the variables in all the dimensions). **(B)** the **quality of variables** (representation of each variable in the 7 dimensions included in the study; it is normalized between 0 and 1, values near 0 indicate a poor representation of the variable in the computed dimensions and the values near 1 indicate a strong representation of the variable in the computed dimensions). **(C,F,I)** coordinates of centers in the seven dimensions (values are normalized between −1 and 1 following a Chi^2^ metric). **(D,G,J)** the relative inertia represents the inertia of each variable in each dimension (normalized between 0 and 1 which represents all the information of a variable in all the dimensions). **(E,H,K)** the quality of variables -*cosine*^2^- represents the distribution of the inertia of each variable across the seven dimensions; it is normalized between 0 and 1 which represents the total inertia of the variable in all the dimensions). Values shown in percentages represent the proportion of relative inertia or quality of each functional configuration with respect all the variables. M1, primary motor cortex; S1, somatosensory cortex; Put, putamen; GPe, external globus pallidumN; STN, subthalamic nucleus; GPi, internal globus pallidum; SN, substantia nigral; MTal, motor thalamus; MTal.

The eigenvalues which represent the relative relevance of each dimension to the total inertia decreased from the first to the seventh dimension. The sum of eigenvalues is 1, and it progressively decreased from 0.28 in the first dimension to 0.08 in the seventh dimension (Figure 5A). The following dimensions had an eigenvalue lower than 0.05 and were not considered in this study. Thus, the accumulated inertia, which shows the inertia of each dimension accumulated with the inertia of the lower dimensions (shown in Figure 5A as a percentage of total inertia), increased from 28% (dimension 1) to 94% (dimension7). It is generally assumed that the number of dimensions analyzed by MCA methods should be high enough to include almost all the information concerning the significant interactions, but avoiding weak relationships which are highly influenced by noise. Thus, dimensions with an eigenvalue lower than 0.05 are often not considered. On the other hand, an accumulated inertia higher than 90 is often enough to detect the main interactions of a system. Seven dimensions were included in the present study because they contained more than 90% of all the information (the seventh dimension showed an accumulated inertia of 94; the value 100 indicates all the information fluxing in the networks), with the eigenvalue of the dimension seven being higher than 0.05. Thus, only the first seven dimensions which contained 94% of all the information were considered in this study (Figure 5B).

GPe, STN, GPi, and SN had similar coordinates in dimension 1 (between +0.6 and +0.7 in a Chi^2^ metric whose values are between −1 and +1) (Figure 5I). This was a marked association which contained 91% of the relative inertia of dimension 1 (the dimension that contained most of the global inertia) (Figure 5J).

The relative inertia represents the inertia of each dimension normalized between 0 and 1 (the value 1 indicates all the information of a variable in all the dimensions). The relative inertia of each of these variables in dimension 1 was around 0.2, and the addition of the relative inertia of GPe, STN, GPi, and SN in this dimension was 0.91, thus showing that most inertia of dimension 1 (91%) is associated to the interaction of these BG. This, and the fact that dimension 1 contained most of the global inertia, suggest that the GPe, STN, GPi, and SN functional interactions are the most relevant in BG, a fact also supported by the finding that these interactions represent 51% of the quality of these variables (Figure 5K). The quality of a variable (which represents the distribution of its inertia across dimensions and was normalized between 0 and 1 here; cosine^2^) was around 0.9 for any of these four centers in dimension 1. The outliers represent the centers that contribute most to the information of each dimension. These results and the fact that GPe, STN, GPi, and SN have the highest coordinate values in this dimension show that these centers are the outliers of dimension 1. Because these centers are the deepest nuclei of BG, their functional aggregation will be referred to as **inner functional configuration** (Ifc).

Dimension 2 grouped M1 and S1, centers which are closely located in coordinates between +0.7 and +0.8 (Figure 5C). The association of these outliers contained 89% of the relative inertia of dimension 2 (Figure 5D) and 61% of the quality (cosine^2^) (Figure 5E) of these variables. The functional aggregation of these centers will be referred to as **synchronic functional configuration** (Sfc).

Dimension 3 grouped Put and MTal (Figure 5F) in coordinates between +0.4 and +0.7. This association contained 77% of the relative inertia of dimension 3 (Figure 5G) and 40% of the quality (Figure 5H) of these variables. Because these are key centers for the interchange of information between BG and the brain cortex, their aggregation will be referred to as **input/output functional configuration** (IOfc).

Dimension 4 grouped the following four centers: Put, MTal, GPe, and STN (Figures 5F,I). These centers were subgrouped in opposite poles, the Put and STN in positive coordinates (between +0.3 and +0.4) and the MTal and GPe in negative coordinates (around −0.4). This association contained 90% of the relative inertia of dimension 4 (Figures 5G,J) and 18% of the quality of these variables (Figures 5H,K). The relatively low value of the quality of this group is justified by the parallel involvement of its centers in other BG configurations. This BG aggregation will be referred to as **input functional configuration** (Ifc).

Dimension 5 showed MTal and GPe (Figures 5F,I) in opposite poles, the MTal in positive coordinates (+0.5) and GPe in negative coordinates (−0.6). This association contained 72% of the relative inertia (Figures 5G,J) of dimension 5 and 29% of the quality of these variables (Figures 5H,K). This aggregation will be referred to as **output functional configuration** (Ofc).

Dimension 6 showed M1 and S1 in opposite poles (Figure 5C), the S1 in positive coordinates (around +0.5) and M1 in negative coordinates (around −0.5). This association contained 68% of the relative inertia (Figure 5D) of dimension 6 and 23% of the quality of these variables (Figure 5E). Thus, most of the quality of M1 and S1 (84%) was distributed between the dimensions 2 (61%) and 6 (23%). Because these centers were aggregated in opposite poles of dimension 6, their functional grouping will be referred to as **diachronic cortical functional configuration** (DCfc).

Finally, dimension 7 displayed SN and GPi in opposite poles (Figure 5I), the SN in positive coordinates (+0.4) and the GPi in negative coordinates (−0.5). This association contained 58% of the relative inertia of dimension 7 (Figure 5J) and 21% of the quality of these variables (Figure 5K), which considering the relatively low eigenvalue of this dimension and the involvement of both centers in other network, suggest that the interaction of these centers shown by dimension 7 is weak. Because the main pathways of SN and GPi go to MTal (the main output center of BG) and both centers were observed in opposite poles, this grouping will be referred to as **alternating output functional configuration** (AOfc).

**Figure 6:**
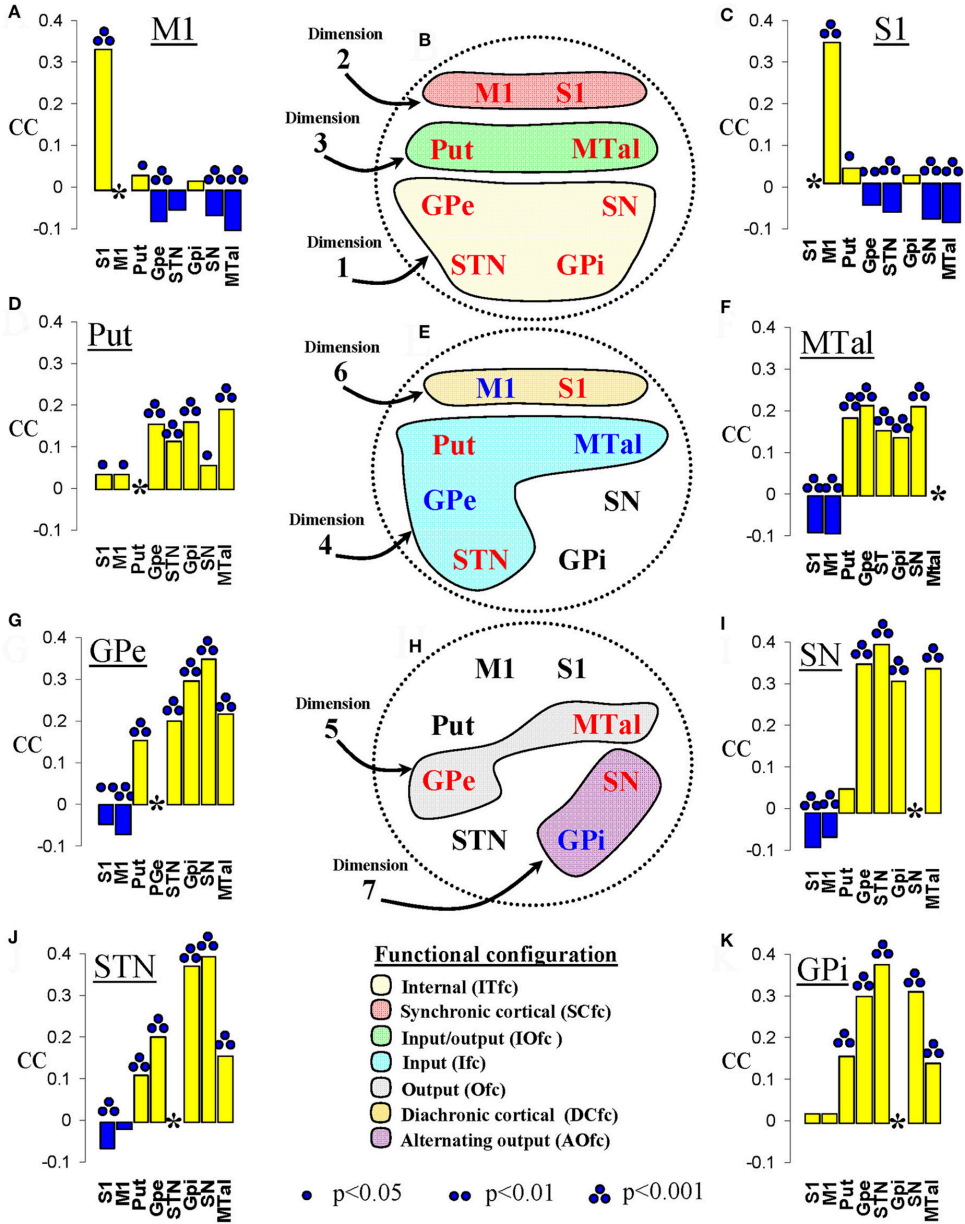
Correspondence coefficient (CC) and functional configurations identified by the multiple correspondence analysis. **(A,C,D,F,G,I–K)** show the CC between a center (underlined at the top of each figure) and all the other centers (indicated at the bottom of each figure). **(B,E,H)** Show the functional configurations identified by the MCA, each with the color indicated at the bottom of the figure and containing the name of the centers involved (in red color for centers found in positive coordinates and in blue color for centers found in negative coordinates). M1, primary motor cortex; S1, somatosensory cortex; Put, putamen; GPe, external globus pallidumN; STN, subthalamic nucleus; GPi, internal globus pallidum; SN, substantia nigral; MTal, motor thalamus; MTal.

Thus, seven BG functional configurations were identified by MCA, one per dimension (see Figures 6B,E,H). Although all these configurations showed enough inertia and quality to be clearly identified, some of them present a more marked influence on the global dynamic of BG than others (ITfc, SCfc, and IOfc had most of the relative inertia of the dimensions with the highest eigenvalues). Another interesting fact was finding different BG in more than one functional configuration, which is particularly relevant in the case of the MTal, this center was functionally linked to three different aggregations (IOfc, Ifc, and Ofc).

The analysis of the Burt table with the CC showed relevant relationships between some particular BG. M1 and S1 showed a positive CC between both and between each of them and the Put. M1 and S1 showed a negative CC between with all the other BG except for the GPi (Figures 6A,C). Put showed a positive CC with all the other BG and, to a lesser but significant degree, with the S1 and M1 (Figure 6D). MTal showed a positive CC with all the other BG and a negative CC with M1 and S1 (Figure 6F). Similarly, the GPe (Figure 6G), SN (Figure 6I), and STN (Figure 6J) showed a positive CC with all the other BG and a negative CC with S1 and M1. Put showed a positive CC with all the other BG and a not significant CC with S1 and M1 (Figure 6K).

## Discussion

MCA of categorized BOLD-signals recorded under resting-state conditions proved to be useful for identifying functional configurations in the motor circuit of BG, even when these centers showed evident non-linear interactions. This unsupervised procedure identified seven configurations, SCfc and DCfc (including M1 and S1), IOfc (Put and MTal), ITfc (GPe, STN, GPi, and SN), Ifc (MTal, Put, STN, and GPe), Ofc (MTal and GPe), and AOfc (SN and GPi). The CC, Pearson and Spearman correlations, and multiple linear regression provided additional information which was useful to understand the interaction of these centers.

### Some methodological considerations

MCA is an extension of principal component analysis whose origin can be traced to the early work of Karl Pearson at the beginning of the twentieth century and whose modern version came from the 1960s in France (*analyse des données* of Jean-Paul BenzécRi). It is also known as reciprocal averaging, optimal scaling, optimal scoring and homogeneity analysis and, as far as we know, it has never been used to study the functional connectivity of the brain with fMRI data. MCA exhibited some advantages over methods commonly used to study cortical networks but whose value decreases when studying centers massively inter-connected by closed-loop feed-back circuits such as those of BG. Because the time-resolution of MRI is 1–2 s, and most neuronal interactions occur with millisecond latencies, the usefulness of fcMRI to study high-speed neuronal interactions is limited. However, very-slow BOLD fluctuations are also useful to identify the centers involved in a network (Filippov, 2005; Fox and Raichle, 2007; Fox et al., 2009; Rayshubskiy et al., 2014), with the cross-correlation of very-slow BOLD fluctuations being the most extensively used method (Biswal et al., 1995). This and other analogous procedures identify a network‘s centers as those showing in-phase fluctuations with a center initially selected as the key member of the network (“seed” center) (Reshef et al., 2011; Su et al., 2013; Kinney and Atwal, 2014). Since only center couples are computed at the same time (a candidate center vs. the seed center), the multi-link between three or more centers cannot be detected, which limits the sensitivity of these methods to study networks with massive interconnections. An important feature of MCA is that its multivariate nature can reveal complex interactions between a group of centers which cannot be detected by pair-wise comparisons. In addition, correlation methods are hypothesis-driven procedures whose results are dependent on the examiner's expectations, which bias the finding of new networks and leaving previously unsuspected networks undiscovered. The ICA (Goebel et al., 2006; Meindl et al., 2010), clustering analysis (Li et al., 2009), and fuzzy (Windischberger et al., 2003) or hierarchical (Cordes et al., 2002) clustering analysis are data-driven methods able to identify networks without using prior information or assumed models. MCA is similar to principal component analysis but whereas this method extracts the variables explaining the largest amount of variance in the data set, the focus of MCA is to examine the associations among variables, which is essentially the aim in this study (to find associations among BG). Cluster analysis discovers whether different variables are related to one other, whereas MCA goes a step further to explain how variables (BG) are related. In addition, these multivariate techniques work with analogical BOLD-oscillations often containing non-linear components which may decrease their sensitivity (Mckeown and Sejnowski, 1998). MCA is less vulnerable to the effect of noise and non-linear activity because it works with categorical data (analogic BOLD-signals were replaced by discrete values representing the high-activity vs. low-activity status), and not with the continuous analogic BOLD-values. This is similar to that performed by digital devices which substitute analogic signals (which are more vulnerable to noisy fluctuations and non-linear influences) by discrete values (0 when analogic values are under a threshold level and 1 when they are over a threshold level). Thus, MCA categorized BOLD activity in two states, the low-states representing the lowest values of the BOLD signal which are presumably associated to a low metabolic rate and neuronal activity, and a high-state which may represent high metabolic rate and neuronal activity. What the MCA approach does is identify the time-coincidence of low-low and high-high status between centers and of low-high and high-low status between centers. In other words, the MCA approach identifies centers whose activity coincides in time (which are involved in the same functional network) and centers whose activity never coincides in time (which are involved in alternative networks never recruited at the same time). Similarly, other methods also use the BOLD time-coincidence to identify the centers of the functional networks, but often working with the analogical data of the BOLD signal. At the end of the process all the functional connectivity methods arrive at a “binary” conclusion regarding the centers (they are activated together and belong to the same network or they are not activated together and belong to different networks). A difference between MCA and other functional connectivity methods is that MCA binarization takes place at the beginning of the process (thus decreasing the influence of noise and non-linear behavior on the identification of networks) whereas binarization in most of the other methods occurs at the end of the process and when conclusions are being made (“being or not being” centers of a network). In addition, MCA is a non-parametric statistic and does not make any distributional assumptions (Greenacre, 1992, 2010; Grassi and Visentin, 1994; Caceres et al., 2010; Pinti et al., 2010). These advantages could be particularly relevant when the centers of the network present a complex non-linear dynamic as that found in BG (see Figure 4). In any case, it could be of interest to compare the behavior of other multivariate methods (e.g., ICA and clustering techniques) with that of MCA, a comparison that could be better performed in data obtained from brain regions simpler than BG and where the expected results could be more evident (there is no obvious procedure to quantify the relative selectivity and sensitivity of each method). As MCA is not an inferential method, the additional use of inferential procedures (such as the CC proposed here and the Pearson and Spearman correlation and the multiple linear regression) may provide complementary information to understand the nature of the functional networks.

### Functional meaning of MCA results

Certain key features can help to interpret the factorial maps provided by MCA. Points near the origin (coordinates near to 0), or those with a low relative inertia or quality, have an undifferentiated profile and do not essentially contribute to the information contained in a particular dimension. Points with both a high absolute coordinate value and a high contribution to the dimension (relative inertia) can be considered as suitable outliers to interpret the meaning of the dimension. Points of a cloud (group of BG in this study) situated far from the origin but close to each other (in similar positive or negative coordinates) present in-phase fluctuations of their functional status (with their high and low status coinciding in time), and have similar profiles of interaction with the other centers (in-phase with centers located in the same positive or negative axis and in anti-phase with centers located in regions of the dimension with the opposite sign). Points of a cloud situated far from the origin but in opposite poles of the axis (positive vs. negative coordinates of the dimension) present anti-phase fluctuations between one another and opposing interaction with the other centers. Bearing these considerations in mind, MCA showed seven functional configurations in BG, a term which indicates the link between their On or Off activity, but which does not necessary imply their co-activation (there could be a time-association of the activation of a center and the deactivation of another) or the existence of direct wiring between them (they can be functional linked by other centers).

### The inner functional configuration (Ifc)

The highest inertia (information) was found in dimension 1, which showed an eigenvalue two-fold higher than any of the other dimensions. GPe, STN, GPi, and SN were grouped together in a small region of dimension 1. Because of their marked spatial proximity and because together they accounted for more than 90% of the relative inertia of the dimension 1, these centers can be considered as members of a single configuration (IFc) and as outliers of the dimension. The marked proximity of IFc centers in the same pole of the axis (positive coordinates) suggests that the four centers have in-phase fluctuations of their functional high/low status, and a similar profile of interaction with the other centers. This possibility was also supported by the CC and the correlation methods, which showed a high positive CC, and high Pearson and Spearman coefficients between the four Ifc centers. These facts also suggest a marked in-phase oscillation of the four centers. Although the Put-MTal and M1-S1 were also grouped in dimension 1, neither their low coordinates nor their low relative inertia and quality, suggest that these groupings are outliers of dimension 1. Put and MTal were located in the same pole (positive), and M1 and S1 in the opposite pole (negative), which suggests that GPe, STN, GPi, and SN have an in-phase fluctuation with Put and MTal, and an anti-phase fluctuation with M1 and S1. This possibility was also suggested by the CC, and the Pearson and Spearman correlations, which showed positive coefficients between the four IFc centers and the MTal and Put, and negative coefficients between the four IFc centers and M1 or S1. This congruency did not always reach statistical value because these tests do not have the same sensitivity (the disagreements of the Pearson correlation are probably due to its lower sensitivity for non-linear interactions). In any case, M1/S1 and MTal/Put “clouds” were later found to be key centers of the functional configurations identified in dimensions 2 and 3 respectively, where their respective groupings presented a much higher inertia and could be better typified. The equations provided by multiple linear regression also agreed with the MCA results (bottom Figure 4). However, these equations do not segregate the interaction of each center in each dimension which could explain some of the disagreements. Although GPe-STN and SN-Put had negative partial correlations which cannot be explained by the interaction of these centers in the Ifc, they could be justified by their interaction in the functional configurations which will be commented on below.

In summary, dimension 1 identified a functional configuration composed of four centers (GPe, STN, GPi, and SN) which fluctuated in phase with MTal and Put and in anti-phase with M1 and S1. The Ifc was found in the dimension with the highest inertia, which suggests that GPe-STN-GPi-SN interactions within this functional configuration prevail over the other interactions which most of these centers presented in other dimensions and functional configurations (e.g., in dimensions 4, 5, and 7). Because the four centers of the Ifc are those located deepest in the BG circuit, their functional aggregation in this dimension was referred to as “internal functional configuration.” The position of GPe/STN/GPi/SN in dimension 1 suggests that the deepest BG (Ifc) act in direct synchrony with the input/output centers of BG (Put/MTal) and in opposite synchrony with the cortical areas involved in the motor circuit (M1/S1). It goes without saying that the interactions of these centers in the other functional configurations identified by MCA also need to be considered.

### The synchronic (SCfc) and diachronic (DCfc) cortical functional configurations

M1 and S1 were grouped in dimension 2 where both cortical areas were located in positive coordinates (SCfc), and dimension 6 where S1 was found in positive and M1 in negative coordinates (DCfc). M1 and S1 were clearly outliers of dimension 2, with coordinates near +0.8 and high values of relative inertia (≈90%) and quality (≈60%). The close proximity of M1 and S1 indicates their in-phase fluctuation and suggests a similar interaction of both cortical centers with BG. The in-phase fluctuation of M1 and S1 was also supported by CC, Pearson and Spearman correlations, and the partial correlation (B in multiple linear regressions). The location of Put in positive regions of dimension 2 suggests that this BG has in-phase oscillations with both cortical areas, and this possibility is also supported by CC, Pearson, and Spearman correlations and the partial correlation.

On the other hand, the location of M1 and S1 in opposite poles of dimension 6 suggests that these cortical areas may also present a slight anti-phase activity. This antagonistic behavior was not reflected in the results provided by the other methods which clearly showed the in-phase M1-S1 relationship expected according to SCfc but not the anti-phase relationship expected according to DCfc. This difference is associated to the higher eigenvalue of dimension 2 with respect to dimension 6, together with the high relative inertia (≈90%) and quality (≈90%) of SCfc compared to the relative inertia (≈68%) and quality (≈23%) of DCfc. As commented above, MCA considers all interactions at the same time, segregating different interactions in different dimensions, a fact that increases the sensitivity of MCA compared to other methods based on pair-wise associations which only detect the predominant relationships. The multiple regression equations suggest that the opposing coordinates of M1 and S1 in dimension 6 can be associated to their different interactions with the GPe, STN, and MTal. Thus, the regression equations showed an inverse relationship of M1 with GPe and of S1 with STN and MTal, differences which were slight (B < 0.7) but significant, and which may be the basis for DCfc.

Thus, M1 and S1 seem to be involved in two different functional configurations, one showing an in-phase hard-interaction (SCfc) and the other showing an anti-phase weak-interaction (DCfc). The weak interaction undetected by CC and correlation methods could be identified by multiple linear regression because this method studies the interaction between individual centers after isolating them from the influence of the other centers (the regression coefficient B in the regression equation represents the partial correlation of each independent variable with the dependent variable after controlling for all the other independent variables). Thus, multiple regression may be a suitable complementary approach for the *post-hoc* analyzing of interactions identified by MCA. The positive B-value in the M1 and S1 regression equations suggests that these cortical centers are frequently co-activated (perhaps by means of massive cortico-cortical connections). The positive B-value of Put in the S1 and M1 regression equations suggests that both cortical centers are co-activated with the Put (perhaps by means of the massive cortico-putaminal pathway whereby cortical information arrives at BG). The inverse relationship observed between the M1 and S1 and most BG suggests that cortical and subcortical networks may be recruited in an alternating way, with the M1/S1 processing (perhaps in connection with other cortical structures) and sending information to the Put in the first step, and then waiting (low-activity status) until the BG return their response to the cortex across the MTal. As commented above, this possibility was also suggested by the ITfc of dimension 1. In this way, the M1/S1 and BG could present alternating activations similar to those reported for M1/S1 and other cortical regions such as those involved in the brain's default network (Fox and Raichle, 2007; Buckner et al., 2008; Raichle, 2009).

### The input (Ifc), output (Ofc) and input/output (IOfc) functional configurations

Dimension 3 grouped the main input (Put) and output (MTal) centers of BG (IOfc). Both centers were located together in the positive pole of dimension 3, suggesting their in-phase co-activation. This possibility is also supported by the CC, the correlation methods and the partial correlation of multiple regression. Put was the outlier of dimension 3, where it had the highest coordinate, relative inertia and quality values. Put displayed a clear in-phase association to MTal (both with positive coordinates) and a less evident anti-phase association with STN, GPi, and SN (with negative coordinates) in the IOfc. The relative inertia and quality was much higher for Put and MTal than for STN, GPi, and SN, suggesting that the Put-MTal in-phase link has a clear role in IOfc, and that the Put vs. STN/GPi/SN anti-phase link is weak or irrelevant. The possible anti-phase associations between Put and STN/GPi/SN cannot be clarified by correlation methods because these centers are also involved in other configurations (MTal in Ifc and Ofc; STN in Ifc; GPi in AOfc; SN in AOfc), a distinction which is not considered in these other methods.

Ifc showed the MTal and GPe in negative coordinates and the Put and STN in positive coordinates. This distribution suggests an anti-phase relationship between the output (MTal projections to the cortex) and input (cortical projections to the Put and STN) centers of BG. GPe showed an anti-phase link with the two other centers of the indirect pathway (see Figure 1A). The inhibitory projections which connect these centers (GABAergic projections from Put to GPe and from GPe to STN) could explain these anti-phase links, the high activity of Put decreasing the GPe activity and, in a second step, increasing the STN activity (now released from the GPe inhibition). However, as can be seen in Figure 1B, Put, GPe and STN display a complex inter-connectivity which, together with the possible “indirect” linking of BG by “crossing centers,” guard against simplistic explanatory hypothesis. The functional connectivity between GPe and MTal is an example of how simple explanations based on the structural connections of centers are not always appropriate. These centers showed an in-phase link in dimension 4 (both were in the negative pole) and an anti-phase link in dimension 5 (MTal in the positive and GPe in the negative pole), even though there are no significant pathways connecting these centers. GPe and MTal showed positive CC and correlation coefficients, which could be linked to their in-phase involvement in the Ifc. In this case, the anti-phase GPe-MTal relationship indicated by the Ofc was undetected by the correlation procedures and CC, methods which working with pair-wise associations cannot identify the simultaneous (and perhaps competitive) interactions between multiple centers (the intensive interaction obscuring slight interactions). Although multiple regression may help to characterize the interaction between individual BG, the efficacy of the partial correlation computed by this method decreases when some centers of the network are not included in the equation (partial correlations are only “controlled” by the other independent variables of the equation), and when the centers present multiple non-linear interactions. The MTal is an example of this fact, showing multiple non-linear associations in different functional configurations (IOfc, Ifc, Ofc). The non-linearity of MTal interactions was shown by both the distribution of residuals of the multiple linear regression, and by the fact that the Spearman coefficient (less sensitive than the parametric correlation but not influenced by non-linearity) was always much higher than the Pearson coefficient (whose high sensitivity decreases in non-linear relationships) (see MTal in Figure 3). The partial correlation of multiple linear regression is more sensitive than both correlation methods but, because it also works with pair-wise associations, it cannot identify multiple interactions (particularly when they present non-linear components) as the MCA do. MCA can identify groups of multiple interacting centers in functional configurations even when interactions present non-linear components. CC (together with multiple regression and the Spearman correlation methods) are useful to estimate the interaction between the components of configurations identified by MCA.

### The alternating output functional configurations (AOfc)

ACfc showed a functional GPi-SN link (ACfc) which accounted for 58% of the inertia of dimension 7 (Figure 5J) and 20% of the quality of all centers (Figure 5K). Bearing in mind that this dimension had the lowest eigenvalue, the AOfc should be considered as representing a weak (but significant) functional association between GPi and SN. The location of their coordinates (positive for SN and negative for GPi) suggests that these centers may be activated in an alternating way (recruited in anti-phase), displaying some different interactions with the other BG. SN and GPi showed some differences in the CC (Figures 6I,K) and in the Pearson and Spearman correlations (Figure 3) with S1 and M1 (negative relationships for the SN and no significant linking for GPi), suggesting a different functional connectivity of GPi and SN with the somatosensory and motor cortex. Although GPi and SN are frequently considered as a single entity in BG models (Albin et al., 1989; Alexander and Crutcher, 1990; Delong, 1990; Tanibuchi et al., 2009), there are biochemical (Windels et al., 2000; Kliem et al., 2007, 2010), odological (Deniau et al., 1982; Nakanishi et al., 1991; Parent et al., 1999; Mailly et al., 2003), and electrophysiological (Wichmann et al., 1999; Kaneda et al., 2005; Kliem et al., 2010; Lafreniere-Roula et al., 2010) data suggesting functional differences between them (Chastan et al., 2009; Nambu, 2011).

### The functional organization of BG according to the MCA

The high complexity of BG interactions is often condensed into simple models that facilitate the understanding of the BG behavior. These models often assume that BG are wired by closed-loop circuits whose excitatory/inhibitory pathways process the incoming cortical information and return it to the brain cortex. Due to methodological restrictions, BG models are mainly based on animal data extrapolated to the human brain. However, there are marked differences between the human and animal BG which hamper the data extrapolation. In addition, a number of subcortical loops and feed-forward circuits little known in humans are often not appropriately included in BG models. All these facts, together with the non-linear complex behavior of their centers, are hampering getting to the bottom of the human BG dynamic, and these obstacles began to be overcome with the use of new imaging methods and analytical procedures (Fox and Raichle, 2007; Shimony et al., 2009; Smith et al., 2009). In the case of BG, the analysis of the BOLD-signal of ROIs of their main centers with non-linear multivariable methods may help to unravel the functional network of these centers in humans. MCA of BOLD-signals have obvious limitations but also clear advantages which have facilitated the study of the functional configurations of the motor-loop of the human BG. MCA is a visualization tool which has been successfully applied in many areas such as medicine, psychology, sociology and geology but which is based on the study of plots and not on statistical tests (the significance of association is tested by the Chi-square test but this test provides no information as to which the significant individual associations are between row-column pairs of the contingence table). Thus, MCA shows the variables (BG) that are related but not what their relationship is. Because MCA dimensions are empirically derived, the validation of BG networks proposed here needs new studies aimed at replicating present data and at supporting their interpretation. Although the interaction between BG has clear non-linear components, linear methods working with continuous analogic data have also proved to be highly sensitive to identifying functional interactions between BG. Thus, to more fully understand the nature of functional connectivity of brain regions, MCA should be used in conjunction with these methods rather than in place of them.

## Conclusions

In summary, evidence is provided here of non-linear components in the functional interaction of most BG, a fact not always considered in BG models. Evidence is also provided showing massive interaction between most BG, a fact suggested by structural data but whose actual relevance in the human brain is difficult to assess by traditional methods. MCA, complemented by CC, correlation methods and the multiple linear regression, showed seven functional configurations of the BG here. Many BG operate in different configurations, suggesting that each BG may perform different functions depending on the network involved, and that networks more than centers are the basic unit of BG activity. Basic questions about the functional configurations of BG need specific studies. Are these seven functional configurations alternating ways for BG activity (each configuration being recruited in a serial way) or could they act in parallel (with some centers being simultaneously engaged to different configurations)? Are the functional configurations of BG dependent on the task which is being performed or are all configurations continuously activated in any circumstance? How do the BG configurations change in Parkinson's disease and other BG disorders? Some of these questions are presently being considered in our laboratory. The initial aim of this study was to test whether MCA could be used to study functional connectivity of brain centers during resting. Thus, MCA was applied to BOLD images of a complex network (BG) obtained during resting in non-selected persons which may represent the healthy population. Present studies in our laboratory are considering possible changes of MCA connectivity associated to aging, sex, BG damage and the task performed during the BOLD recording. In addition, MCA may be applied to the full parceling of the cortical surface, which allows the study of other cortico-subcortical networks of BG. The methodological development of MCA application to imaging studies could facilitate the application of MCA to all boxels of brain images, which could be particularly useful in the study of cortical networks and for comparing MCA with other functional connectivity methods.

## Author contributions

CR was the main organizer of the study, being involved in the planning of the study, analysis and interpretation of data and in the manuscript review. She also participated in data recordings. IM participated in data recordings. AS participated in data recordings. MR was involved in the planning of the study, analysis and interpretation of data and in the manuscript review.

### Conflict of interest statement

The authors declare that the research was conducted in the absence of any commercial or financial relationships that could be construed as a potential conflict of interest.

## References

[B1] Albin R. L. Young A. B. Penney J. B. (1989). The functional anatomy of basal ganglia disorders. Trends Neurosci. 12, 366–375. 10.1016/0166-2236(89)90074-X 2479133

[B2] Alexander G. E. Crutcher M. D. (1990). Functional architecture of basal ganglia circuits: neural substrates of parallel processing. Trends Neurosci. 13, 266–271. 10.1016/0166-2236(90)90107-L 1695401

[B3] Alexander G. E. Delong M. R. Strick P. L. (1986). Parallel organization of functionally segregated circuits linking basal ganglia and cortex. Annu. Rev. Neurosci. 9, 357–381. 10.1146/annurev.ne.09.030186.002041 3085570

[B4] Almeida R. M. Infantosi A. F. Suassuna J. H. Costa J. C. (2009). Multiple correspondence analysis in predictive logistic modelling: application to a living-donor kidney transplantation data. Comput. Methods Programs Biomed. 95, 116–128. 10.1016/j.cmpb.2009.02.003 19328584

[B5] Ambrogi F. Biganzoli E. Boracchi P. (2005). Multiple correspondence analysis in S-PLUS. Comput. Methods Programs Biomed. 79, 161–167. 10.1016/j.cmpb.2005.03.001 15975690

[B6] Anderson J. S. Druzgal T. J. Lopez-Larson M. Jeong E. K. Desai K. Yurgelun-Todd D. (2011). Network anticorrelations, global regression, and phase-shifted soft tissue correction. Hum. Brain Mapp. 32, 919–934. 10.1002/hbm.21079 20533557 PMC3220164

[B7] Avolio M. Montagnoli S. Marino M. Basso D. Furia G. Ricciardi W.. (2013). Factors influencing quality of life for disabled and nondisabled elderly population: the results of a multiple correspondence analysis. Curr. Gerontol. Geriatr. Res. 2013:258274. 10.1155/2013/258274 23878536 PMC3710593

[B8] Ayele D. Zewotir T. Mwambi H. (2014). Multiple correspondence analysis as a tool for analysis of large health surveys in African settings. Afr. Health Sci. 14, 1036–1045. 10.4314/ahs.v14i4.35 25873942 PMC4386317

[B9] Biswal B. Yetkin F. Z. Haughton V. M. Hyde J. S. (1995). Functional connectivity in the motor cortex of resting human brain using echo-planar MRI. Magn. Reson. Med. 34, 537–541. 10.1002/mrm.1910340409 8524021

[B10] Bouilland S. Loslever P. (1998). Multiple correspondence analysis of biomechanical signals characterized through fuzzy histograms. J. Biomech. 31, 663–666. 10.1016/S0021-9290(98)00054-2 9796689

[B11] Buckner R. L. Andrews-Hanna J. R. Schacter D. L. (2008). The brain's default network: anatomy, function, and relevance to disease. Ann. N.Y. Acad. Sci. 1124, 1–38. 10.1196/annals.1440.011 18400922

[B12] Caceres A. Basagana X. Gonzalez J. R. (2010). Multiple correspondence discriminant analysis: an application to detect stratification in copy number variation. Stat. Med. 29, 3284–3293. 10.1002/sim.3890 21170921

[B13] Chastan N. Westby G. W. Yelnik J. Bardinet E. Do M. C. Agid Y.. (2009). Effects of nigral stimulation on locomotion and postural stability in patients with Parkinson's disease. Brain 132, 172–184. 10.1093/brain/awn294 19001482

[B14] Cole M. W. Pathak S. Schneider W. (2010). Identifying the brain's most globally connected regions. Neuroimage 49, 3132–3148. 10.1016/j.neuroimage.2009.11.001 19909818

[B15] Cordes D. Haughton V. Carew J. D. Arfanakis K. Maravilla K. (2002). Hierarchical clustering to measure connectivity in fMRI resting-state data. Magn. Reson. Imaging 20, 305–317. 10.1016/S0730-725X(02)00503-9 12165349

[B16] Costa P. S. Santos N. C. Cunha P. Cotter J. Sousa N. (2013). The use of multiple correspondence analysis to explore associations between categories of qualitative variables in healthy ageing. J. Aging Res. 2013:302163. 10.1155/2013/302163 24222852 PMC3810057

[B17] Damoiseaux J. S. Rombouts S. A. Barkhof F. Scheltens P. Stam C. J. Smith S. M.. (2006). Consistent resting-state networks across healthy subjects. Proc. Natl. Acad. Sci. U.S.A. 103, 13848–13853. 10.1073/pnas.0601417103 16945915 PMC1564249

[B18] Delong M. R. (1990). Primate models of movement disorders of basal ganglia origin. Trends Neurosci. 13, 281–285. 10.1016/0166-2236(90)90110-V 1695404

[B19] Deniau J. M. Kitai S. T. Donoghue J. P. Grofova I. (1982). Neuronal interactions in the substantia nigra pars reticulata through axon collaterals of the projection neurons. An electrophysiological and morphological study. Exp. Brain Res. 47, 105–113. 10.1007/BF00235891 6288427

[B20] Di Martino A. Scheres A. Margulies D. S. Kelly A. M. Uddin L. Q. Shehzad Z.. (2008). Functional connectivity of human striatum: a resting state FMRI study. Cereb. Cortex 18, 2735–2747. 10.1093/cercor/bhn041 18400794

[B21] Filippov I. V. (2005). Very slow brain potential fluctuations (<0.5 Hz) in visual thalamus and striate cortex after their successive electrical stimulation in lightly anesthetized rats. Brain Res. 1066, 179–186. 10.1016/j.brainres.2005.10.061 16324687

[B22] Fox M. D. Raichle M. E. (2007). Spontaneous fluctuations in brain activity observed with functional magnetic resonance imaging. Nat. Rev. Neurosci. 8, 700–711. 10.1038/nrn2201 17704812

[B23] Fox M. D. Snyder A. Z. Vincent J. L. Raichle M. E. (2007). Intrinsic fluctuations within cortical systems account for intertrial variability in human behavior. Neuron 56, 171–184. 10.1016/j.neuron.2007.08.023 17920023

[B24] Fox M. D. Zhang D. Snyder A. Z. Raichle M. E. (2009). The global signal and observed anticorrelated resting state brain networks. J. Neurophysiol. 101, 3270–3283. 10.1152/jn.90777.2008 19339462 PMC2694109

[B25] Fox P. T. Raichle M. E. (1986). Focal physiological uncoupling of cerebral blood flow and oxidative metabolism during somatosensory stimulation in human sujacts. Proc. Natl. Acad. Sci. U.S.A. 83, 1140–1144. 10.1073/pnas.83.4.1140 3485282 PMC323027

[B26] Fox P. T. Raichle M. E. Mintun M. A. Dence C. (1988). Nonoxidative glucose consumption during focal physiologic neural activity. Science 241, 462–464. 10.1126/science.3260686 3260686

[B27] Goebel R. Esposito F. Formisano E. (2006). Analysis of functional image analysis contest (FIAC) data with brainvoyager QX: from single-subject to cortically aligned group general linear model analysis and self-organizing group independent component analysis. Hum. Brain Mapp. 27, 392–401. 10.1002/hbm.20249 16596654 PMC6871277

[B28] Gopinath K. Ringe W. Goyal A. Carter K. Dinse H. R. Haley R.. (2011). Striatal functional connectivity networks are modulated by fMRI resting state conditions. Neuroimage 54, 380–388. 10.1016/j.neuroimage.2010.07.021 20637878

[B29] Grassi M. Visentin S. (1994). Correspondence analysis applied to grouped cohort data. Stat. Med. 13, 2407–2425. 10.1002/sim.4780132306 7701143

[B30] Greenacre M. (1992). Correspondence analysis in medical research. Stat. Methods Med. Res. 1, 97–117. 10.1177/096228029200100106 1341654

[B31] Greenacre M. (2010). Correspondence analysis of raw data. Ecology 91, 958–963. 10.1890/09-0239.1 20462111

[B32] Guinot C. Latreille J. Morizot F. Ambroisine L. Mauger E. Tenenhaus M.. (2002). Assessment of sun reactive skin type with multiple correspondence analysis, hierarchical and tree-structured classification methods. Int. J. Cosmet. Sci. 24, 207–216. 10.1046/j.1467-2494.2002.00140.x 18498512

[B33] Haber S. N. (2003). The primate basal ganglia: parallel and integrative networks. J. Chem. Neuroanat. 26, 317–330. 10.1016/j.jchemneu.2003.10.003 14729134

[B34] Jo H. J. Gotts S. J. Reynolds R. C. Bandettini P. A. Martin A. Cox R. W. (2013). Effective preprocessing procedures virtually eliminate distance-dependent motion artifacts in resting State FMRI. J. Appl. Math. 1, 1–9. 10.1155/2013/935154 PMC3886863 24415902

[B35] Kaneda K. Tachibana Y. Imanishi M. Kita H. Shigemoto R. Nambu A.. (2005). Down-regulation of metabotropic glutamate receptor 1alpha in globus pallidus and substantia nigra of parkinsonian monkeys. Eur. J. Neurosci. 22, 3241–3254. 10.1111/j.1460-9568.2005.04488.x 16367790

[B36] Kim S. G. Ugurbil K. (1997). Comparison of blood oxygenation and cerebral blood flow effects in fMRI: estimation of relative oxygen consumption change. Magn. Reson. Med. 38, 59–65. 10.1002/mrm.1910380110 9211380

[B37] Kinney J. B. Atwal G. S. (2014). Equitability, mutual information, and the maximal information coefficient. Proc. Natl. Acad. Sci. U.S.A. 111, 3354–3359. 10.1073/pnas.1309933111 24550517 PMC3948249

[B38] Kliem M. A. Maidment N. T. Ackerson L. C. Chen S. Smith Y. Wichmann T. (2007). Activation of nigral and pallidal dopamine D1-like receptors modulates basal ganglia outflow in monkeys. J. Neurophysiol. 98, 1489–1500. 10.1152/jn.00171.2007 17634344

[B39] Kliem M. A. Pare J. F. Khan Z. U. Wichmann T. Smith Y. (2010). Ultrastructural localization and function of dopamine D1-like receptors in the substantia nigra pars reticulata and the internal segment of the globus pallidus of parkinsonian monkeys. Eur. J. Neurosci. 31, 836–851. 10.1111/j.1460-9568.2010.07109.x 20374284 PMC4305335

[B40] Lafreniere-Roula M. Kim E. Hutchison W. D. Lozano A. M. Hodaie M. Dostrovsky J. O. (2010). High-frequency microstimulation in human globus pallidus and substantia nigra. Exp. Brain Res. 205, 251–261. 10.1007/s00221-010-2362-8 20640411

[B41] Lee K. Tak S. Ye J. C. (2011). A data-driven sparse GLM for fMRI analysis using sparse dictionary learning with MDL criterion. IEEE Trans. Med. Imaging 30, 1076–1089. 10.1109/TMI.2010.2097275 21138799

[B42] Li K. Guo L. Nie J. Li G. Liu T. (2009). Review of methods for functional brain connectivity detection using fMRI. Comput. Med. Imaging Graph. 33, 131–139. 10.1016/j.compmedimag.2008.10.011 19111443 PMC2724763

[B43] Mailly P. Charpier S. Menetrey A. Deniau J. M. (2003). Three-dimensional organization of the recurrent axon collateral network of the substantia nigra pars reticulata neurons in the rat. J. Neurosci. 23, 5247–5257. 12832549 10.1523/JNEUROSCI.23-12-05247.2003 PMC6741183

[B44] Marceglia S. Foffani G. Bianchi A. M. Baselli G. Tamma F. Egidi M.. (2006). Dopamine-dependent non-linear correlation between subthalamic rhythms in Parkinson's disease. J. Physiol. (Lond). 571, 579–591. 10.1113/jphysiol.2005.100271 16410285 PMC1805793

[B45] Mchaffie J. G. Stanford T. R. Stein B. E. Coizet V. Redgrave P. (2005). Subcortical loops through the basal ganglia. Trends Neurosci. 28, 401–407. 10.1016/j.tins.2005.06.006 15982753

[B46] Mckeown M. J. Sejnowski T. J. (1998). Independent component analysis of fMRI data: examining the assumptions. Hum. Brain Mapp. 6, 368–372. 9788074 10.1002/(SICI)1097-0193(1998)6:5/6<368::AID-HBM7>3.0.CO;2-E PMC6873375

[B47] Meindl T. Teipel S. Elmouden R. Mueller S. Koch W. Dietrich O.. (2010). Test-retest reproducibility of the default-mode network in healthy individuals. Hum. Brain Mapp. 31, 237–246. 10.1002/hbm.20860 19621371 PMC6871144

[B48] Meyer N. Ferlicot S. Vieillefond A. Peyromaure M. Vielh P. (2004). Contribution of multiple correspondence analysis in histopathology. Ann. Pathol. 24, 149–160. 10.1016/S0242-6498(04)93938-7 15220834

[B49] Murphy K. Birn R. M. Handwerker D. A. Jones T. B. Bandettini P. A. (2009). The impact of global signal regression on resting state correlations: are anti-correlated networks introduced? Neuroimage 44, 893–905. 10.1016/j.neuroimage.2008.09.036 18976716 PMC2750906

[B50] Nakanishi H. Kita H. Kitai S. T. (1991). Intracellular study of rat entopeduncular nucleus neurons in an *in vitro* slice preparation: response to subthalamic stimulation. Brain Res. 549, 285–291. 10.1016/0006-8993(91)90469-C 1909205

[B51] Nambu A. (2011). Somatotopic organization of the primate Basal Ganglia. Front. Neuroanat. 5:26. 10.3389/fnana.2011.00026 21541304 PMC3082737

[B52] Obeso J. A. Marin C. Rodriguez-Oroz C. Blesa J. Benitez-Temino B. Mena-Segovia J.. (2008a). The basal ganglia in Parkinson's disease: current concepts and unexplained observations. Ann. Neurol. 64(Suppl. 2), S30–S46. 10.1002/ana.21481 19127584

[B53] Obeso J. A. Rodriguez-Oroz M. C. Benitez-Temino B. Blesa F. J. Guridi J. Marin C.. (2008b). Functional organization of the basal ganglia: therapeutic implications for Parkinson's disease. Mov. Disord. 23(Suppl. 3), S548–S559. 10.1002/mds.22062 18781672

[B54] Oldfield R. C. (1971). The assessment and analysis of handedness: the Edinburgh inventory. Neuropsychologia 9, 97–113. 10.1016/0028-3932(71)90067-4 5146491

[B55] Parent A. (1990). Extrinsic connections of the basal ganglia. Trends Neurosci. 13, 254–258. 10.1016/0166-2236(90)90105-J 1695399

[B56] Parent M. Levesque M. Parent A. (1999). The pallidofugal projection system in primates: evidence for neurons branching ipsilaterally and contralaterally to the thalamus and brainstem. J. Chem. Neuroanat. 16, 153–165. 10.1016/S0891-0618(99)00008-3 10422736

[B57] Penney J. B. Jr. Young A. B. (1986). Striatal inhomogeneities and basal ganglia function. Mov. Disord. 1, 3–15. 10.1002/mds.870010102 2848190

[B58] Pinti A. Rambaud F. Griffon J. L. Ahmed A. T. (2010). A tool developed in Matlab for multiple correspondence analysis of fuzzy coded data sets: application to morphometric skull data. Comput. Methods Programs Biomed. 98, 66–75. 10.1016/j.cmpb.2009.09.009 19892428

[B59] Power J. D. Mitra A. Laumann T. O. Snyder A. Z. Schlaggar B. L. Petersen S. E. (2014). Methods to detect, characterize, and remove motion artifact in resting state fMRI. Neuroimage 84, 320–341. 10.1016/j.neuroimage.2013.08.048 23994314 PMC3849338

[B60] Raichle M. E. (1998). Behind the scenes of functional brain imaging: a historical and physiological perspective. Proc. Natl. Acad. Sci. U.S.A. 95, 765–772. 10.1073/pnas.95.3.765 9448239 PMC33796

[B61] Raichle M. E. (2009). A paradigm shift in functional brain imaging. J. Neurosci. 29, 12729–12734. 10.1523/JNEUROSCI.4366-09.2009 19828783 PMC6665302

[B62] Rayshubskiy A. Wojtasiewicz T. J. Mikell C. B. Bouchard M. B. Timerman D. Youngerman B. E.. (2014). Direct, intraoperative observation of ~0.1 Hz hemodynamic oscillations in awake human cortex: implications for fMRI. Neuroimage 87, 323–331. 10.1016/j.neuroimage.2013.10.044 24185013 PMC3961585

[B63] Redgrave P. Marrow L. Dean P. (1992). Topographical organization of the nigrotectal projection in rat: evidence for segregated channels. Neuroscience 50, 571–595. 10.1016/0306-4522(92)90448-B 1279464

[B64] Rennie T. W. Roberts W. (2009). Data mining of tuberculosis patient data using multiple correspondence analysis. Epidemiol. Infect. 137, 1699–1704. 10.1017/S0950268809002787 19450381

[B65] Reshef D. N. Reshef Y. A. Finucane H. K. Grossman S. R. Mcvean G. Turnbaugh P. J.. (2011). Detecting novel associations in large data sets. Science 334, 1518–1524. 10.1126/science.1205438 22174245 PMC3325791

[B66] Rodriguez M. Muniz R. Gonzalez B. Sabate M. (2004). Hand movement distribution in the motor cortex: the influence of a concurrent task and motor imagery. Neuroimage 22, 1480–1491. 10.1016/j.neuroimage.2004.02.040 15275905

[B67] Rodriguez M. Pereda E. Gonzalez J. Abdala P. Obeso J. A. (2003a). How is firing activity of substantia nigra cells regulated? Relevance of pattern-code in the basal ganglia. Synapse 49, 216–225. 10.1002/syn.10233 12827640

[B68] Rodriguez M. Pereda E. Gonzalez J. Abdala P. Obeso J. A. (2003b). Neuronal activity in the substantia nigra in the anaesthetized rat has fractal characteristics. Evidence for firing-code patterns in the basal ganglia. Exp. Brain Res. 151, 167–172. 10.1007/s00221-003-1442-4 12768261

[B69] Rodriguez-Sabate C. Llanos C. Morales I. Garcia-Alvarez R. Sabate M. Rodriguez M. (2015). The functional connectivity of intralaminar thalamic nuclei in the human basal ganglia. Hum. Brain Mapp. 36, 1335–1347. 10.1002/hbm.22705 25429921 PMC6869176

[B70] Rodriguez-Sabate C. Sabate M. Llanos C. Morales I. Sanchez A. Rodriguez M. (2016). The functional connectivity in the motor loop of human basal ganglia. Brain Imaging Behav. 11, 417–429. 10.1007/s11682-016-9512-y 26935555

[B71] Saad Z. S. Gotts S. J. Murphy K. Chen G. Jo H. J. Martin A.. (2012). Trouble at rest: how correlation patterns and group differences become distorted after global signal regression. Brain Connect. 2, 25–32. 10.1089/brain.2012.0080 22432927 PMC3484684

[B72] Sagawa Y. Jr. Armand S. Lubbeke A. Hoffmeyer P. Fritschy D. Suva D.. (2013). Associations between gait and clinical parameters in patients with severe knee osteoarthritis: a multiple correspondence analysis. Clin. Biomech. 28, 299–305. 10.1016/j.clinbiomech.2013.01.008 23410553

[B73] Schroll H. Hamker F. H. (2013). Computational models of basal-ganglia pathway functions: focus on functional neuroanatomy. Front. Syst. Neurosci. 7:122. 10.3389/fnsys.2013.00122 24416002 PMC3874581

[B74] Selemon L. D. Goldman-Rakic P. S. (1985). Longitudinal topography and interdigitation of corticostriatal projections in the rhesus monkey. J. Neurosci. 5, 776–794. 2983048 10.1523/JNEUROSCI.05-03-00776.1985 PMC6565017

[B75] Shimony J. S. Zhang D. Johnston J. M. Fox M. D. Roy A. Leuthardt E. C. (2009). Resting-state spontaneous fluctuations in brain activity: a new paradigm for presurgical planning using fMRI. Acad. Radiol. 16, 578–583. 10.1016/j.acra.2009.02.001 19345899 PMC2818666

[B76] Smith S. M. Fox P. T. Miller K. L. Glahn D. C. Fox P. M. Mackay C. E.. (2009). Correspondence of the brain's functional architecture during activation and rest. Proc. Natl. Acad. Sci. U.S.A. 106, 13040–13045. 10.1073/pnas.0905267106 19620724 PMC2722273

[B77] Su L. Wang L. Shen H. Feng G. Hu D. (2013). Discriminative analysis of non-linear brain connectivity in schizophrenia: an fMRI Study. Front. Hum. Neurosci. 7:702. 10.3389/fnhum.2013.00702 24155713 PMC3804761

[B78] Su X. Wijayasinghe C. S. Fan J. Zhang Y. (2016). Sparse estimation of Cox proportional hazards models via approximated information criteria. Biometrics 72, 751–759. 10.1111/biom.12484 26873398 PMC4982849

[B79] Tanibuchi I. Kitano H. Jinnai K. (2009). Substantia nigra output to prefrontal cortex via thalamus in monkeys. I. Electrophysiological identification of thalamic relay neurons. J. Neurophysiol. 102, 2933–2945. 10.1152/jn.91287.2008 19692504

[B80] Touso M. M. Popolin M. P. Crispim Jde A. Freitas I. M. Rodrigues L. B. Yamamura M.. (2014). Social stigma and the families of patients with tuberculosis: a study based on cluster and multiple correspondence analysis. Cien. Saude Colet. 19, 4577–4586. 10.1590/1413-812320141911.46062013 25351323

[B81] Treserras S. Boulanouar K. Conchou F. Simonetta-Moreau M. Berry I. Celsis P.. (2009). Transition from rest to movement: brain correlates revealed by functional connectivity. Neuroimage 48, 207–216. 10.1016/j.neuroimage.2009.06.016 19527788

[B82] Van Dijk K. R. Hedden T. Venkataraman A. Evans K. C. Lazar S. W. Buckner R. L. (2010). Intrinsic functional connectivity as a tool for human connectomics: theory, properties, and optimization. J. Neurophysiol. 103, 297–321. 10.1152/jn.00783.2009 19889849 PMC2807224

[B83] Wichmann T. Bergman H. Starr P. A. Subramanian T. Watts R. L. Delong M. R. (1999). Comparison of MPTP-induced changes in spontaneous neuronal discharge in the internal pallidal segment and in the substantia nigra pars reticulata in primates. Exp. Brain Res. 125, 397–409. 10.1007/s002210050696 10323285

[B84] Windels F. Bruet N. Poupard A. Urbain N. Chouvet G. Feuerstein C.. (2000). Effects of high frequency stimulation of subthalamic nucleus on extracellular glutamate and GABA in substantia nigra and globus pallidus in the normal rat. Eur. J. Neurosci. 12, 4141–4146. 10.1046/j.1460-9568.2000.00296.x 11069610

[B85] Windischberger C. Barth M. Lamm C. Schroeder L. Bauer H. Gur R. C.. (2003). Fuzzy cluster analysis of high-field functional MRI data. Artif. Intell. Med. 29, 203–223. 10.1016/S0933-3657(02)00072-6 14656487

